# Structural Analysis of Disk Resonance Gyroscope

**DOI:** 10.3390/mi8100296

**Published:** 2017-09-30

**Authors:** Dunzhu Xia, Lingchao Huang, Lei Xu, Haiyu Gao

**Affiliations:** Key Laboratory of Micro-Inertial Instrument and Advanced Navigation Technology, Ministry of Education, School of Instrument Science and Engineering, Southeast University, Nanjing 210096, China; 220162744@seu.edu.cn (L.H.); 220152697@seu.edu.cn (L.X.); 220142659@seu.edu.cn (H.G.)

**Keywords:** disk resonator gyroscope (DRG), quality factor (*Q*), finite element method (FEM), frequency split

## Abstract

In this paper, we present two design methods to improve the performance of disk resonator gyroscope (DRG), including decreasing the frequency split and increasing the quality factor (*Q*). The structure parameters, which can affect the frequency split and *Q* value were concluded with the help of the FEM software. Meanwhile, devices with different parameters were designed, fabricated, and tested, and the experimental result was in accordance with the simulation. With the proposed methods, the DRG was selected with a high *Q* value and a low frequency split to satisfy the demand of high performance. The weakness and future works were pointed at last.

## 1. Introduction

The micro-mechanical vibration gyroscope is developing with the improvement of the micro-electro-mechanical system (MEMS) technology, which results in the smaller size, lower cost, and higher performance devices. Hence, it widely used in a variety of consumer electronics applications [1].

As one of the potential devices that can achieve high performance, the disk resonator gyroscope (DRG) has attracted more and more attention in recent years. It can meet the demand for threshold compact northfinding applications and can be integrated in inertial navigation systems [2].

In order to improve the performance of MEMS gyroscopes, several methods including temperature control, electrostatic tuning, and optimal design were presented [3,4,5,6,7,8]. With these methods, the polysilicon DRG that was implemented through the epitaxial silicon encapsulation process and had a *Q* value of 50 k at a resonant frequency of 264 kHz was reported in [5]. Later, the <100> silicon gyroscope of similar geometry and process is presented in [6], the *Q* value of it was larger than 100 k on *n* = 3 modes at a resonant frequency of 70 kHz, and the mean frequency split of it was 21 Hz. A ring gyroscope operated at the trefoil mode (*n* = 3) with a *Q* value of 10 k in vacuum and a mean frequency split ratio of 225 ppm at 130 kHz is achieved in [7]. The DRG with a *Q* value of ~1.3 M and 40 ppm frequency split at 2.745 MHz is presented in [8]. On the whole, it can be seen that all of the methods focus on increasing the frequency split and decreasing the *Q* value.

According to Reference [9], if the gyroscope operates at the mode-matched condition, which means the frequency split is too small and can be ignored, the gyroscope will have the perfect symmetry in drive and sense modes, and the rotation-induced Coriolis signal is amplified by the *Q* value of the sense mode. Therefore, the higher *Q* value means a higher sensitivity in the gyroscope. Meanwhile, a higher *Q* value also represents a longer decay time, higher resolution, higher SNR (signal-to-noise ratio), and lower energy dissipation. The relation between resonance frequency and the *Q* value of resonance gyroscope can be approximately expressed as [10]:

(1) $$Q=\frac{f}{\Delta f_{-3db}}$$

where *f* is the resonance frequency and Δ*f_−_*_3*db*_ is the −3*db* bandwidth.

Hence, the *Q* value of DRG is an important performance index in structure design and devices with a high *Q* value can be achieved by the optimal design of structures and an improved manufacturing process. *Q* value is mainly limited by a variety of energy loss mechanisms, such as thermoelastic damping (*Q_TED_*), support loss (*Q_support_*), and air damping (*Q_air_*), which can be expressed as:

(2) $$Q=\frac{1}{Q_{TED}}+\frac{1}{Q_{support}}+\frac{1}{Q_{air}}+\cdot \cdot \cdot$$

From Equation (2), it is concluded that the most important thing to improve *Q* value is finding the main energy loss mechanisms and the effects in it.

Beside the *Q* value, the frequency split also contributes to the performance of DGR. It is clear that the smaller frequency split can bring a higher sensitivity of the gyroscope when the *Q* value is constant. However, due to the anisotropic material properties of single crystal silicon substrate [11,12] and manufacturing imperfection [1], the frequency split of drive and sense modes is inevitable. In order to minimize the frequency split caused by the material nature, a feasible way is manufacturing DRG by isotropic material such as <111> silicon [13,14] or polysilicon [5,15]. Unfortunately, this approach requires development of new processes for etching, bonding, and packaging, and it is likely to cause more challenges than they can be avoided [9]. Meanwhile, the frequency split of DRG made by isotropic silicon can hardly be adjusted by changing the parameters of structure, which will be proved in the article later.

An alternative method to eliminate the effect of frequency mismatches operates the gyroscope at the *n* = 3 mode condition [1,6,7,16], but the amplitude of *n* = 3 mode is much smaller than the *n* = 2 mode, which means that the sensitivity of the gyroscope will decrease considerably. Also, the *n* = 3 mode can hardly adjust the frequency split through structure design, which is similar to the DRG in <111> silicon. Another way to solve the problem is by compensating the anisotropy of the monocrystalline silicon by design of structure, such as slightly changing the location of spokes, the width of spokes, and rings [17].

In this paper, we presented several methods of structural design based on the model of DRG structure from [17], to compensate for the frequency split caused by the anisotropy of <100> silicon [18]. In reference [17], it mainly focused on the application of spoke location (angle) and spoke width. In this paper, the effect of spoke length, spoke number, and ring number are also introduced and simulated using the finite element method (FEM) software ANSYS in Section 2. Meanwhile, the energy dissipation mechanisms of DRG is analyzed and simulated in Section 3. The fabrication process is described in Section 4. The testing results are presented to compare with the simulation results in Section 5. Finally, the testing results are summarized.

## 2. The Effect of DRG Structure on Frequency Split 

**Figure 1:**
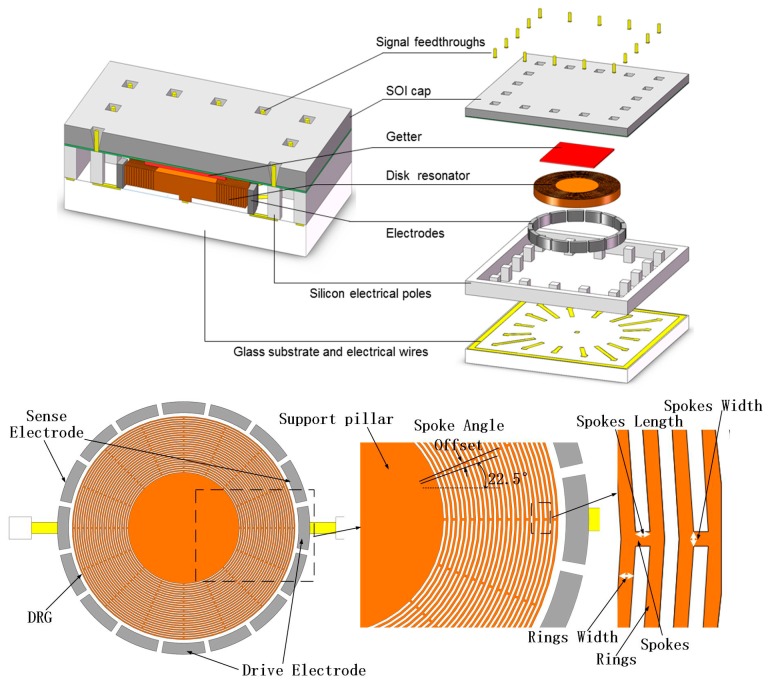
Schematic diagram of disk resonator gyroscope (DRG) in Silicon-On-Insulator (SOI) packaging.

**Figure 2:**
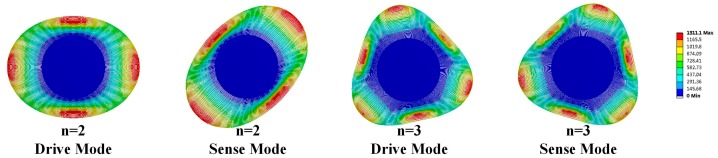
The different modes of DRG.

The structure of DRG consists of a set of multiple concentric rings and electrodes evenly distributed outside of the rings, as shown in Figure 1. Each couple of adjacent rings are connected by the spokes, which are interleaved with an angular offset. The ring resonator is anchored with the support pillar at the center and suspended on the glass substrate. The operational principle of DRG is shown in Figure 2, it is driven into oscillation along the drive axis, and then the Coriolis force caused by the rotation in detecting axis, which is vertical to the DRG plane, gives rise to the motion in its sense axis. The angle between the drive axis and the sense axis is 45° in *n* = 2 mode and 30° in *n* = 3 mode.

For the sake of analysing the structure of the DRG preliminarily, the simplified equations of the motion of the DRG are expressed by [19]:

(3) $$\begin{array}{l} \ddot{x}+2\xi_{x}\omega_{x}\dot{x}+\omega_{x}^{2}x=\frac{F_{0}\sin\left(\omega_{x}t\right)}{m_{eff}}\\ \ddot{y}+2\xi_{y}\omega_{y}\dot{y}+\omega_{y}^{2}y=4A_{g}\Omega \dot{x} \end{array}$$

where *x*, *y* are the displacements of the drive axis and sense axis, *ξ* is the damping ratio, *ω* is the resonant frequency, *m_eff_* is the effective mass, *A_g_* is the angular gain, Ω is the angular rate, and *F*_0_ is the driving force. Thus, the equation of motion in drive axis can be concluded as:

(4) $$x\left(t\right)=\frac{Q_{x}F_{0}}{k_{x}}\sin\left(\omega_{x}t-\frac{\pi }{2}\right)$$

where *Q_x_* is the *Q* value of drive mode, *Q_x_* = 1/2 *ξ_x_*, *k_x_* is the stiffness of the drive axis and *k_x_* = *m_eff_* × *ω*_x_^2^. Put it into Equation (3) and the mechanical sensitivity can be written as:

(5) $$\left|\frac{y\left(t\right)}{\Omega }\right|=\frac{4A_{g}Q_{x}F_{0}}{\omega_{x}m_{eff}^{2}\sqrt{{\left(\omega_{y}^{2}-\omega_{x}^{2}\right)}^{2}+{\left(\frac{\omega_{y}\omega_{x}}{Q_{y}}\right)}^{2}}}$$

According to Equation (5), there are three ways to increase the mechanical sensitivity: (1) increasing the driving force; (2) decreasing the frequency split between drive and sense modes through optimal design of the gyroscope; and, (3) improving the *Q* value in drive and sense modes.

In addition, the effective mass and angular gain is:

(6) $$\begin{array}{c} m_{eff}^{}=\underset{V}{∭}\rho \left(\phi_{x1}^{2}+\phi_{y1}^{2}+\phi_{z1}^{2}\right)dV\\ =\underset{V}{∭}\rho \left(\phi_{x2}^{2}+\phi_{y2}^{2}+\phi_{z2}^{2}\right)dV \end{array}$$

(7) $$A_{g}=\frac{\underset{V}{∭}\rho \left(\phi_{x1}\phi_{y2}-\phi_{x2}\phi_{y1}\right)dV}{2m_{eff}}$$

where (*φ_x_*_1_, *φ_y_*_1_, *φ_z_*_1_, *φ_x_*_2_, *φ_y_*_2_, *φ_z_*_2_) are the shape functions of the disk resonator, and *ρ* is the density of the material [20].

In order to reduce the frequency split, the four approaches including varying the placement of spokes, the width of spokes, the number of spokes, and the width of rings are presented. In other words, the effective stiff could be adjusted to compensate for the anisotropy of <100> silicon through these slight changes in structure. Finally, the effect of the methods are demonstrated under the theoretical analysis and FEM simulation presented in the section.

### 2.1. The Small Offset Angle of Spokes Placement

**Figure 3:**
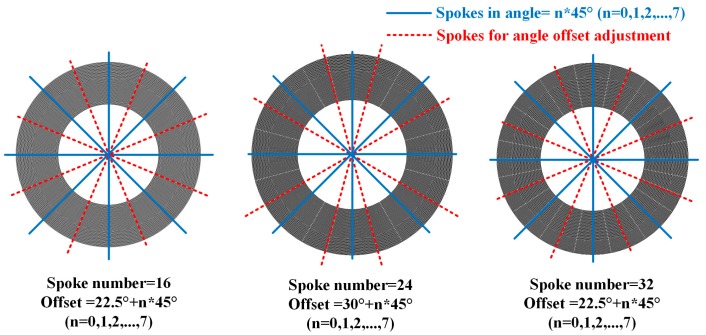
The offset angle locations in DRGs with different spoke number.

The small angular offset of spokes locates is in 22.5° + *n* × 45° (*n* = 0, 1, 2, ..., 7) position when the number of spokes is 16. Similarly, the offset is in 30° + *n* × 45° and 22.5° + *n* × 45° (*n* = 0, 1, 2, ..., 7) when the spoke number is 24 and 32, respectively, as shown in Figure 3. Since the resonator frequency of device is determined by the dimension and position of the rings and support beams, an offset angle adjustment as small as ±(0.1°–0.7°) of spokes position can slightly change the frequency to achieve the mode-matching condition of the resonator.

Taking the material anisotropy of <100> single crystal silicon into consideration, the Young’s modulus is a function of the crystal direction *E(θ)* [21]. Furthermore, considering that the end of the innermost spokes are connected to the central anchor and the other end to ring, the cantilever beam-bending can be used as the model to make the approximate theoretical to analyze and predict the effective stiffness of spokes. Therefore, the model can be expressed as [9,17]:

(8) $$\begin{array}{l} k_{eff,s}=\sum_{i}k_{s}\left(\theta_{i}\right)，\\ k_{s}\left(\theta_{i}\right)=\sum_{i}\frac{3E\left(\theta_{i}\right)I}{{\left(L\sin\left(\theta_{i}\right)\right)}^{3}}=\frac{E\left(\theta_{i}\right)wh^{3}}{4\left(L\sin\left(\theta_{i}\right)\right)} \end{array}$$

where *k_eff,s_* is the effective stiffness of spokes, *I* is the moment of inertia, *θ_i_* is the angle from the principle axes of the *i^th^* spoke, *w*, *h,* and *L* are the width, thickness, and length of spokes.

Owing to the complexity of model analysis, the FEM software ANSYS is used to simulate the model of the resonator when it works in its resonant frequency of *n* = 2 mode. The relationship between the frequency splits and the varying spoke positions is shown in Table 1.

From the data in the table, it is seen that the frequency split of the DRG manufactured in <100> silicon can be adjusted to the level close to 0.1 Hz when it works in *n* = 2 mode.

### 2.2. The Varying Width and Length of Spokes

According to Equation (3), it is obvious that the effective stiffness of spokes is related to the width, thickness, and length of spokes. Since the thickness of spokes is difficult to control during the process of fabrication, the spoke width and length are chosen to adjust the frequency split.

**Figure 4:**
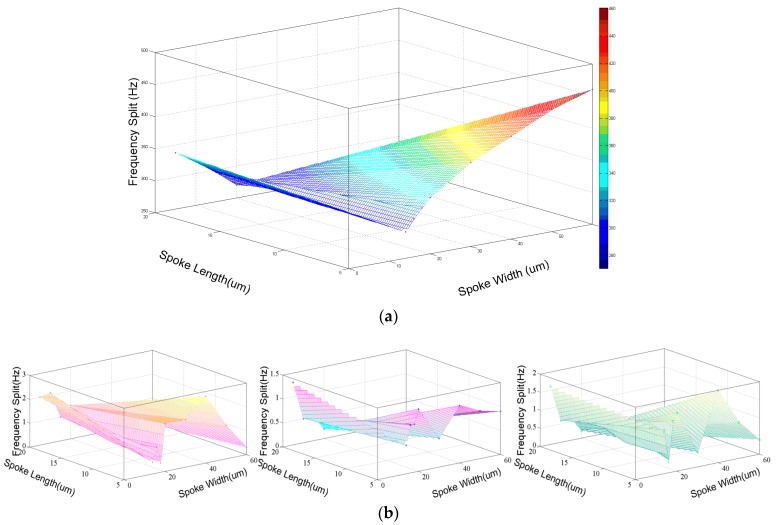
Relationship between the frequency splits and different length and width of spokes: (**a**) <100> DRG in *n* = 2, (**b**) From left to right: <111> DRG in *n* = 2, <100> DRG in *n* = 3, <111> DRG in *n* = 3.

Similarly, the simulation of resonant frequency of *n* = 2 mode is performed with the help of ANSYS, and the frequency splits of different widths of spokes are shown in Figure 4. From the figures, it can be concluded that the frequency splits are positively correlated to the width of spokes and negatively related to the length of spokes in condition of the <100> silicon DRG working in *n* = 2 mode. Besides, the frequency splits of <100> DRG in *n* = 2 mode, <111> silicon DRG in *n* = 2 and *n* = 3 modes showed no obvious relationship with the parameters of spokes.

### 2.3. The Varying Number of Spokes

**Figure 5:**
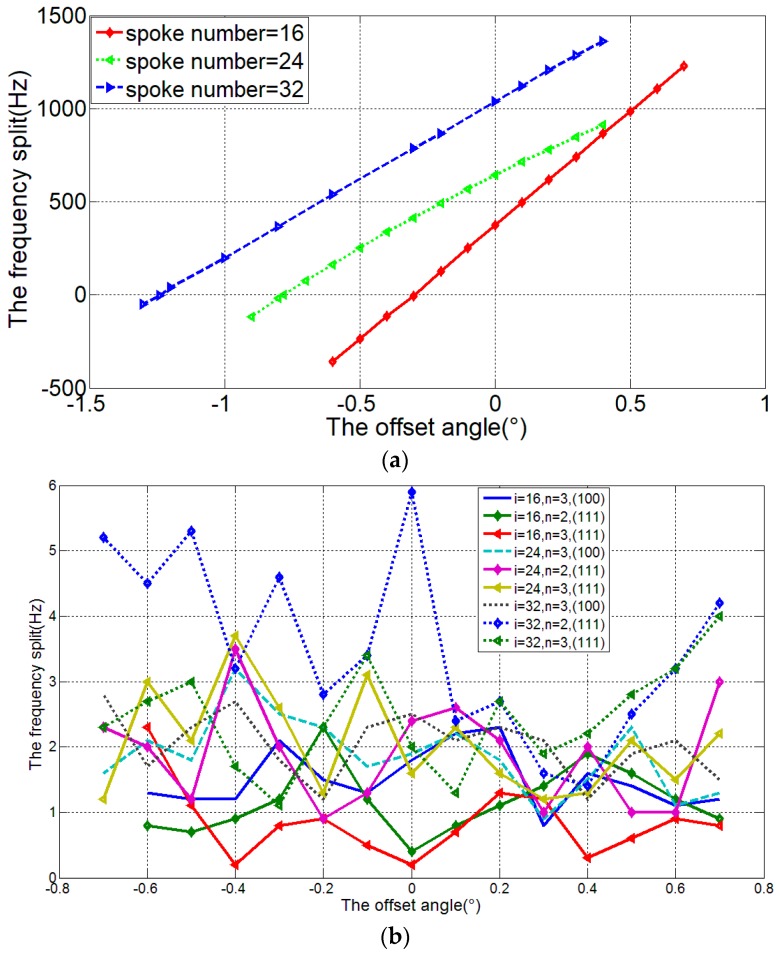
Relationship between the frequency splits and the varying spoke positions (**a**) DRG made by <100> silicon and work in *n* = 2 mode, (**b**) DRG made by <100> silicon and work in *n* = 3 mode; <111> silicon, work in *n* = 2 and *n* = 3 mode.

Also, the effective stiffness can be changed by adjusting the number of spoke *i* when the other factors of the spokes including the position, width, and length remain, as shown in Equation (2). The resonator frequency of *i* = 16, 24, 32 is simulated and the frequency splits of different spoke number and offset angle are shown in Figure 5.

From these figures, it is concluded that the different number of spokes can affect the frequency splits. Meanwhile, for <100> silicon DRG in *n* = 2 mode, the larger spoke number will cause the increasing of offset angle for mode matching.

### 2.4. The Varying Width of Rings

The rings of DRG can be considered as a parallel connection of many double-U-shaped springs, so the effective stiffness of resonator can be expressed as:

(9) $$k=\sum k_{d}=\sum E\left(\theta \right)h\cdot {\left(\frac{w}{L}\right)}^{3}=\sum_{i}\sum_{j}E\left(\theta_{i}\right)h\cdot {\left(\frac{w}{R_{j}\theta_{i}}\right)}^{3}$$

where *k_d_* is the effective stiffness of the double-U-shaped spring, *h* is the thickness of the rings, *R* is the radius of the rings, *w* is the width of the rings, and *θ* is the angular range of the springs.

**Figure 6:**
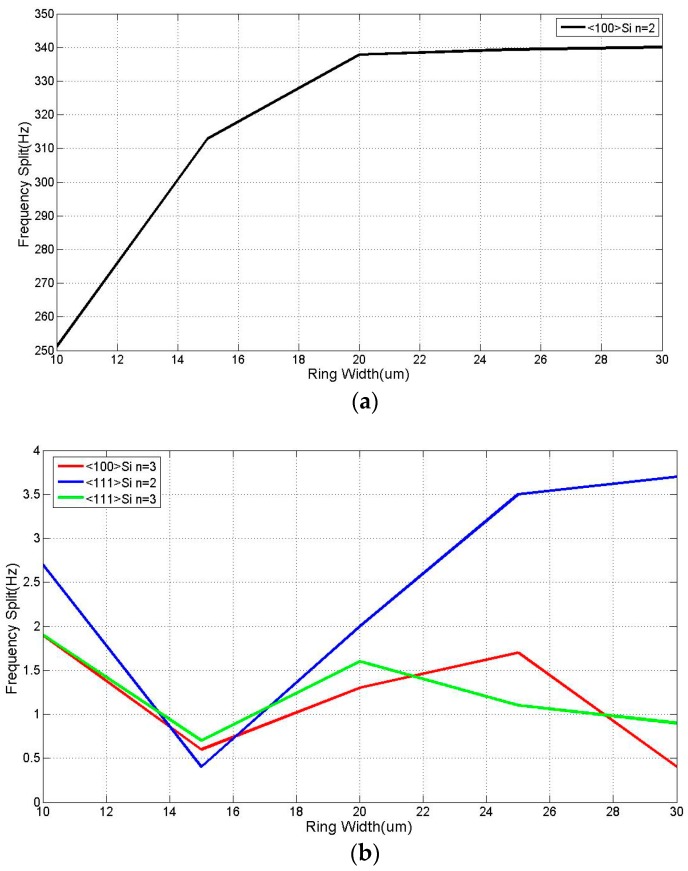
Relationship between the frequency splits and ring width (**a**) DRG made by <100> silicon and work in *n* = 2 mode, (**b**) DRG made by <100> silicon and work in *n* = 3 mode; <111> silicon, work in *n* = 2 and *n* = 3 mode.

According to Equation (9), *k* is proportional to the *w*^3^, so a slight change in the width of rings as much as 1–2 µm will have significant effect on the resonant frequency without changing the operation mode of resonator. According to the simulation result of the resonators, the frequency splits of different widths of different DRG and modes are shown in Figure 6.

From Figure 6a, it can be seen that the frequency has positive correlation with the width of rings, but the correlation becomes weak when the width of ring is over 20 µm. Furthermore, similar to other conditions, the frequency splits of <111> silicon DRG and <100> silicon DRG in *n* = 3 mode is much smaller and can be ignored, as shown in Figure 6b.

## 3. Analysis of Energy Dissipation

From Equation (5), it can be concluded that the *Q* values of drive and sense modes can affect the mechanical sensitivity. According to the simulation results mentioned later, the resonant frequencies of drive and sense modes vary with the *Q* values. Hence, in order to estimate the performance of DRG more correctly, a new evaluation indicator *I_Qf_* is mentioned according to [22]:

(10) $$I_{Qf}=Q_{x}f_{x}Q_{y}f_{y}$$

where *Q_x_* and *Q_y_* are the *Q* values of drive and sense modes, and *f_x_* and *f_y_* are the resonant frequencies of drive and sense modes.

Based on Equation (2), *Q* value is inversely proportional to the energy dissipation, which includes thermoelastic dissipation, support loss, air damping and so on. But as mentioned before, the DRG is packaged in a high vacuum environment, so the air damping can be neglected. With the help of FEA software COMSOL Multiphysics, we can analyze the thermoelastic dissipation and support loss, respectively, to find the methods and improve the *Q* value by structure design.

### 3.1. Thermoelastic Dissipation

When the mechanical resonator operates, it will vibrate at a certain frequency that leads to the variation of strain with the variation of temperature. Meanwhile, the heat current is irreversible, which causes the energy loss called thermoelastic dissipation. For most of the resonance gyroscopes, thermoelastic dissipation is one of the key limitations to the *Q* of gyroscopes [23].

There are closed-form expressions for *Q_TED_* in resonators with simple and ideal geometric features [24]. However, it is very difficult to obtain the analytic solutions of resonators with complex geometries. In addition, due to the imperfect fabrication and the anisotropy of the materials, it is very difficult to build the ideal mathematic model of *Q_TED_*. Therefore, the FEM software, COMSOL Multiphysics, is used to simulate the *Q_TED_* of DRGs with different structure parameters.

**Figure 7:**
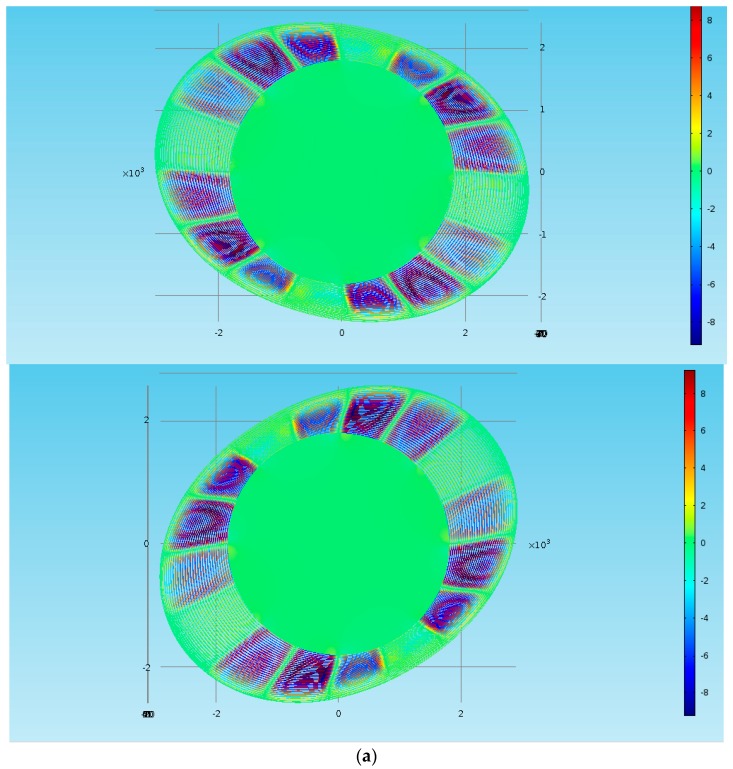

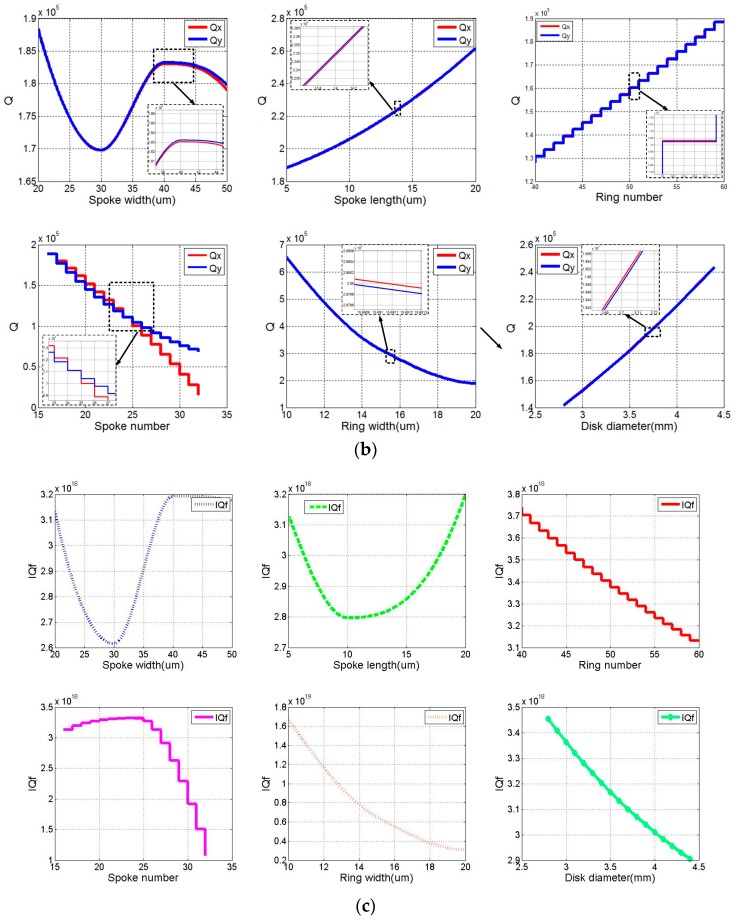
Simulation results in COMSOL Multiphysics. (**a**) Temperature departure of DRG in drive and sense modes (*n* = 2), (**b**) Simulation results of *Q_TED_* in DRG, and (**c**) Simulation results of *I_Qf_* in DRG.

The relationships between the *Q_TED_* of disk resonator and spoke width, spoke length, and other factors are fitted by the simulation results, which is shown as Figure 7.

From Figure 7a, it can be seen that the temperature departure in the rings mainly existed in the part with sever deformation, which means that the adjustment in rings is more effective than spokes to improve *Q_TED_*. Furthermore, based on Figure 7b, we can conclude that the *Q_TED_* is positively correlated with the ring number and spoke length, while it is negatively correlated with the spoke number, ring width and disk diameter. In addition, it is not appropriate to judge the performance without considering the resonant frequency, so the *I_Qf_* is shown in Figure 7c, and its trend is similar to *Q_TED_*. In a word, it can be expressed as:

(11) $$\left\{ \begin{array}{l} Q_{TED}\propto \left(\begin{array}{ccccc} L_{s} & N_{r} & \frac{1}{N_{s}} & \frac{1}{w_{r}} & \frac{1}{d_{D}} \end{array}\right)\\ I_{Qf}\propto \left(\begin{array}{ccc} \frac{1}{N_{s}} & \frac{1}{w_{r}} & \frac{1}{d_{D}} \end{array}\right) \end{array}\right.$$

where *L_s_* is the length of spoke, *N_r_* and *N_s_* are the number of rings and spokes, *w_r_* is the width of ring, and *d_D_* is the diameter of disk. Meanwhile through data analysis, it can be found that the dominant elements are the spoke number, the ring number, and the ring width.

Due to the limitation of the overall dimensions and the required resonant frequency value, it is necessary to get the tradeoff among them by adjusting the structural parameters of DRG. In this case, the number of spokes and rings is set to 16 and 60, rings width is 15 µm, spoke length is 10 µm, spoke width is 20 µm, and disk diameter is 4.4 mm.

### 3.2. Support Loss

As an important dissipation mechanism for mechanical disk resonance, an analysis model of support loss has been built in [25,26,27,28,29,30]. The support stem is generally under the center of disk to eliminate the support damping, but with the micro structure, it is becoming increasingly difficulty to fabricate enough small support stem in the central position. The COMSOL Multiphysics software and the Perfectly Match Layer (PML) are applied to simulate the support damping. The material of DRG is <100> silicon and the supporting base is made by BF33 glass, Table 2 lists the material prosperities of <100> single crystal silicon and BF33 glass.

**Figure 8:**
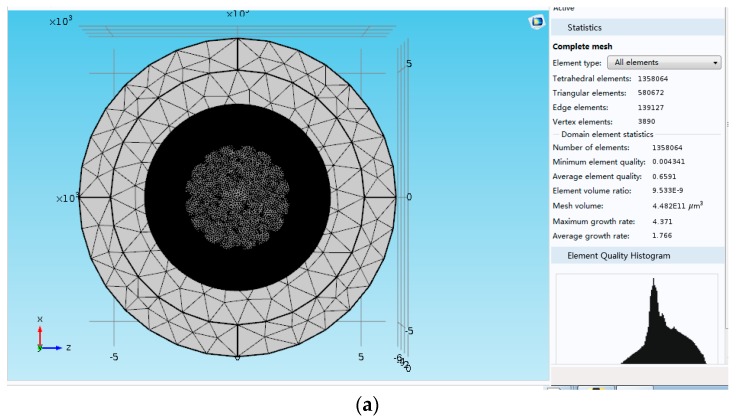

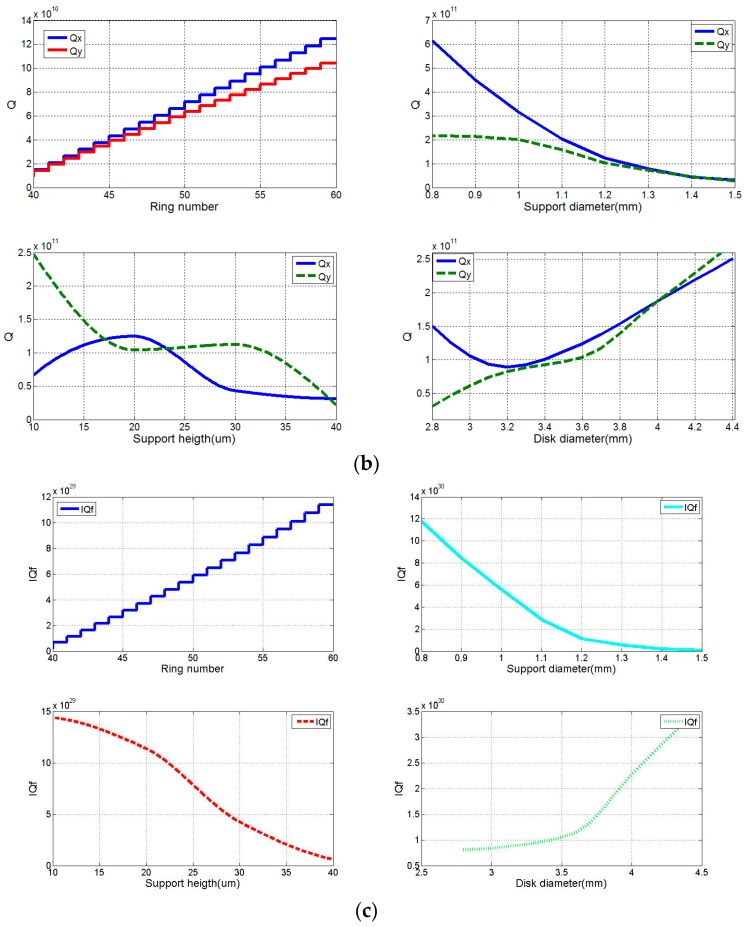
Simulation results of *Q*_support_ in COMSOL Multiphysics: (**a**) The mesh result of DRG for simulation of support loss, (**b**) Simulation results of *Q*_support_ in DRG, and (**c**) Simulation results of *I_Qf_* in DRG.

*Q_support_* greatly depends on the ratio of the support pillar radius and the disk radius. Meanwhile, a quarter wavelength rule exists for the height of the support pillar [23]. Thus, the main structure parameters of DRG which have effect on support loss are the diameter and height of support pillar, the diameter, and the ring number of DRG. Moreover, all the conditions are simulated in the software, and the simulation results are shown in Figure 8.

In Figure 8b, it is difficult to conclude the relationship between *Q_support_* and the structure parameters of support pillar and disk because of the fluctuant connection. However, the trend of *I_Qf_* in support loss is clear, which can be seen in Figure 8c. *I_Qf_* is positively related to the ring number and the diameter of disk. Besides, *I_Qf_* is inverse proportional to the support height and diameter.

Although using the conclusion above can help in designing the DRG with lower support loss, the *Q_support_* is much greater than *Q_TED_*, which means that the support loss is negligible when compared with thermoelastic dissipation. Hence, the parameters, which are set for low thermoelastic dissipation, remain unchanged. Besides, the height and diameter of support pillar are chosen as 20 µm and 0.8 mm.

## 4. Fabrication

**Figure 9:**
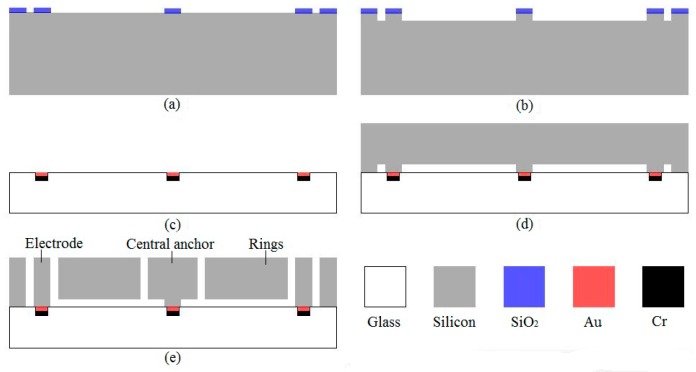
Main process flow of fabrication of DRG.

The DRG of multiple rings can be fabricated by a conventional three-mask silicon on glass (SOG) process. The 60 µm thickness device layer is made on the low resistivity, boron-doped (P-type) <100> single crystal silicon wafer. The borosilicate glass of BF33 is chosen as the material of substrates for its close coefficient of thermal expansion with silicon. The main process flows are shown in Figure 9. First, 2 µm plasma-enhanced chemical vapor deposition (PECVD) SiO_2_ layer is deposited on the back side of silicon wafer and patterned by mask1 to define the anchor (Figure 9a). Then, the central post is formed by the deep reactive ion etching (DRIE), and the gap between the glass substrate and device is created at the same time (Figure 9b). Next, the photoresist are patterned by mask2, and then a 30/300 nm thickness Cr/Au is deposited in the pattern to form the electrical interconnects and pads through lift-off process (Figure 9c). After that, the Si-glass anodic bonding is performed, and then the silicon wafer is thinned with the chemical mechanical polishing (CMP) process (Figure 9d). Finally, 2 µm PECVD SiO_2_ layer is deposited on the front side of silicon wafer and patterned by mask3, and the resonator structure and electrodes are simultaneously released using the Bosch ICP (inductively coupled plasma) process (Figure 9e).

**Figure 10:**
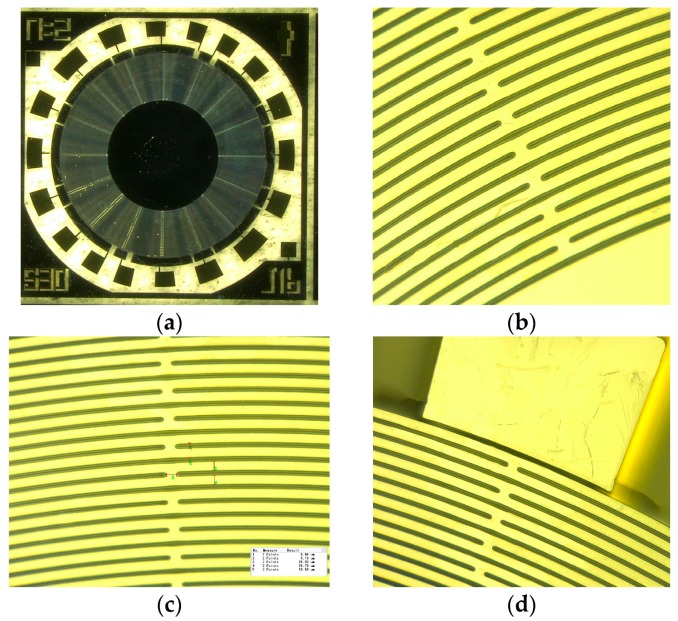
The photo of the fabricated DRG. (**a**) Bird eye view of fabrication disk resonator; (**b**) Zoomed-in the view of rings, spokes and disk; (**c**) Zoomed-in view of rings and spokes; (**d**) Zoomed-in the view of electrodes

The micrographs of DRG fabricated is shown Figure 10, which include the bird eye view of the fabricated resonator (Figure 10a) and the zoomed-in view of rings, spokes and electrodes (Figure 10b–d).

In Figure 10a, *n* = 2 represents that the operating mode of DRG is 2, and J16 shows the number of spokes is 16. Figure 10b–d show the detail of rings and spokes, and ten devices with different design parameters are picked up to analyze the fabricating accuracy. The ring width, spoke width, and spoke length of them are measured, as shown in Figure 10c, and are then compared with the design values to get the fabricating errors. After that, the mean values of these errors are calculated and the mean value of each error in the three parts mentioned above is less than 5%. Therefore, it can be seen that the fabrication result is excellent.

## 5. Experimental Results

**Figure 11:**
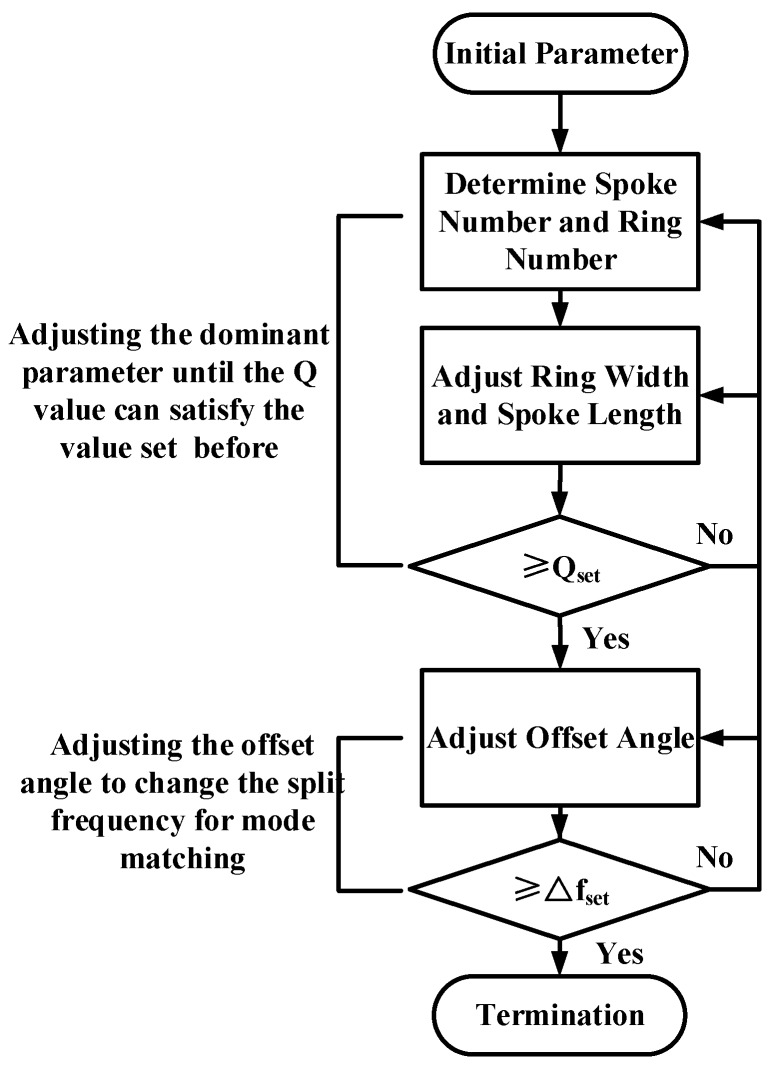
Parameters selection process for DRG.

According to the results mentioned above, the process to find the optimal parameters of the DRG is created and shown in Figure 11.

**Figure 12:**
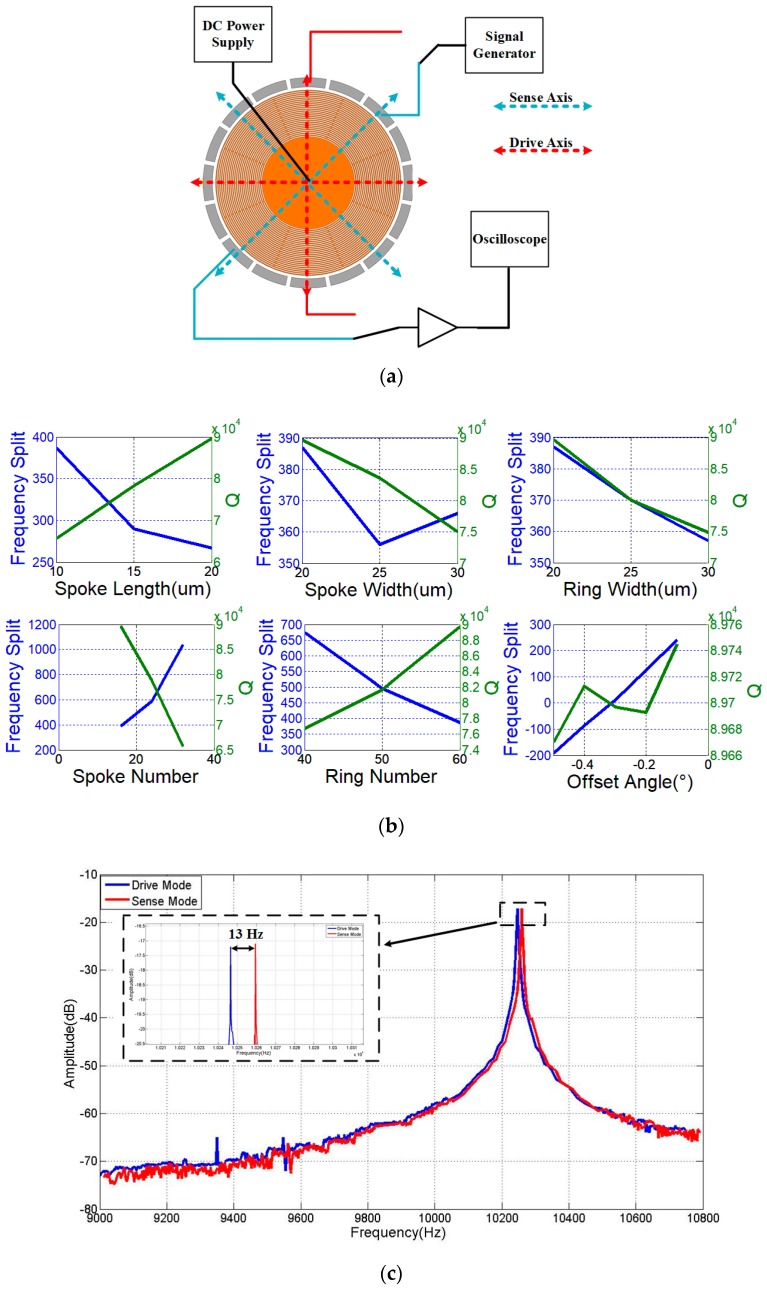
Experimental setup and results: (**a**) Experimental setup for testing the frequency and *Q* of DRG; (**b**) Comparison of experiment results of DRG with different parameters; and, (**c**) The frequency sweeping data of the selected DRG.

The experimental setup is shown in Figure 12a. In the experimental setup the bias voltage is 5 V. Due to the effect of bias voltage, the electrostatic spring softening phenomenon is observed from drive and sense modes, which contributes to the difference between the frequencies of reality and simulation. Since it will strengthen the extra electrostatic spring softening in the specific ground connection, the other electrodes is floating in this experiment. Although the electrostatic spring softening affects the resonant frequency, the frequency difference in different mode is very close when the bias voltage and the design values in two modes are the same. On this account, the influence in frequency split from electrostatic spring softening is ignored in this study. Besides, the device works in a certain vacuum condition (about 1 mTorr) and a controlled temperature of 50 °C.

Figure 12b shows the frequency split and *Q* value of the tested DRGs. The *Q* value here is the mean value of *Q* values in drive and sense modes. The experimental result of devices have difference with the simulation. However, the effects of the structure parameters are in accordance with the simulation. Hence, the conclusions from analysis and simulation are meaningful for design.

Figure 12c shows the frequency sweeping data of the best DRG selected from devices in Figure 12b, and its parameters are shown in Table 3. Meanwhile, it is also the device with optimal parameters based on the simulation. The frequency split and *Q* value of the selected device are 13 Hz and about 90,000, respectively, while the simulation result are 2 Hz and 160,000.

## 6. Conclusions

Frequency split and *Q* value are two important factors for DRG. By analysis and simulation, the dominant parameters to be optimized have an advantageous effect on the frequency split and *Q* value. After that, we designed, fabricated, and tested several disk resonators with different structural parameters, and the experimental data supported the assumptions concluded from the simulation. Based on these, we selected the DRG with a high *Q* value of 90,000 and a low frequency split of 13 Hz in resonant frequency of about 10.2 kHz without any electrostatic tuning technique. However, in our work, many other elements, including the environment effects, the thermo-elastic effects, and so on, are neglected. Hence, the future work will focus on the effects of these factors in frequency split and *Q* value.

## Figures and Tables

**Table 1:** The Results of Simulation of Frequency Splits in DRGs with different material, mode and spoke angular offset.

Number of Spokes	Offset Angle of Spokes (°)	Frequency Splits			
		<100>Si, *n* = 2	<100>Si, *n* = 3	<111>Si, *n* = 2	<111>Si, *n* = 3
16	+0.7	1229.6	1.2	0.9	0.8
	+0.6	1108.5	1.1	1.2	0.9
	+0.5	985.2	1.4	1.6	0.6
	+0.4	864.8	1.6	1.9	0.3
	+0.3	741.2	0.8	1.4	1.2
	+0.2	618.4	2.3	1.1	1.3
	+0.1	497	2.2	0.8	0.7
	0	375.1	1.8	0.4	0.2
	−0.1	252.3	1.3	1.2	0.5
	−0.2	128.3	1.5	2.3	0.9
	−0.3	6.8	2.1	1.2	0.8
	−0.4	−113.7	1.2	0.9	0.2
	−0.5	−236.7	1.2	0.7	1.1
	−0.6	−358.9	1.3	0.8	2.3
24	−0.1	566.5	1.8	2.1	1.6
	−0.2	493.3	2.2	2.6	2.3
	−0.3	413.4	1.9	2.4	1.6
	−0.4	338	1.7	1.3	3.1
	−0.5	254	2.3	0.9	1.3
	−0.6	163	2.5	2	2.6
	−0.7	78	3.2	3.5	3.7
	−0.78	2	1.8	1.2	2.1
	−0.8	−17	2.1	2	3
	−0.9	−116	1.6	2.3	1.2
32	0	1039	2.1	1.6	1.9
	−0.2	867	2.3	2.7	2.7
	−0.3	789	2.1	2.4	1.3
	−0.6	541	2.5	5.9	2
	−0.8	368	2.3	3.4	3.4
	−1	197	1.2	2.8	2.3
	−1.2	41	1.8	4.6	1.1
	−1.24	3	2.7	3.2	1.7
	−1.3	−48	2.3	5.3	3

**Table 2:** The Material Prosperities of <100> Silicon and BF33 Glass.

Prosperity	<100> Silicon	BF33 Glass
Density	2329 kg/m^3^	2200 kg/m^3^
Young’s modulus	130 GPa	64 GPa
Poisson ratio	0.28	0.2
Thermal conductivity	148 W/(m·K)	1.2 W/(m·K)
Coefficient of thermal expansion	2.6 × 10^−6^ K^−1^	3.25 × 10^−6^ K^−1^
Specific heat capacity	700 J/(kg·K)	830 J/(kg·K)

**Table 3:** The Parameters of Selected DRG.

Parameter	Value
Spoke number	16
Ring number	60
Spoke width	20 µm
Spoke length	10 µm
Ring width	20 µm
Offset Angle	−0.3°
Electrode gap	1.5 µm
Support pillar height	20 µm
